# Older adults' perspectives on key domains of childhood social and economic experiences and opportunities: a first step to creating a multidimensional measure

**DOI:** 10.1186/1742-5573-4-14

**Published:** 2007-11-06

**Authors:** Irene H Yen, Anita L Stewart, Teresa Scherzer, Eliseo J Pérez-Stable

**Affiliations:** 1 Medical Effectiveness Research Center for Diverse Populations, Division of General Internal Medicine, Department of Medicine, University of California, San Francisco, CA 94143, USA; 2 School of Nursing, University of California, San Francisco, USA; 3 Institute for Health & Aging, University of California, San Francisco, USA

## Abstract

**Objectives:**

Although research has found that childhood socioeconomic status (SES) is associated with physical and mental health in mid- and later life, most of these studies used conventional, single dimension SES measures for the childhood period such as household income or educational attainment of parents. Life course and health disparities research would benefit from identification and measurement of a variety of childhood social and economic experiences and opportunities that might affect health in later life.

**Design:**

This study utilized qualitative research methods to identify key dimensions of childhood experiences related to SES. We conducted in-depth interviews with 25 adults age 55 to 80 years from diverse economic and ethnic backgrounds. Topics included home, neighborhood, school, and work experiences during early childhood and adolescence. Interviews were audio-taped and transcripts were coded to identify thematic domains.

**Results:**

We identified eight thematic domains, many of which had clear subdomains: home and family circumstances, neighborhood, work and money, potential for advancement through schooling, school quality and content, discrimination, influence and support of adults, and leisure activities. These domains highlight individual characteristics and experiences and also economic and educational opportunities.

**Conclusion:**

These domains of childhood social and economic circumstances add breadth and depth to conventional conceptualization of childhood SES. When the domains are translated into a measurement tool, it will allow for the possibility of classifying people along multiple dimensions, such as from a low economic circumstance with high levels of adult support.

## Introduction

Socioeconomic status (SES) is an important correlate of health with consistent descriptive reports that people in higher SES categories have lower risk for morbidity and mortality than people in lower SES categories [1,2]. As attention increasingly turns to identifying mechanisms of health disparities, the direct role of SES on health is drawing attention [3]. For example, researchers suggest that economic circumstances, social status, social capital, and human capital are possible mechanisms that connect SES to health [3-5]. Beyond identifying the mechanisms that link adult SES to adult health status, researchers are also recognizing the dynamic nature of SES over a lifespan. Life course perspectives are gaining recognition in epidemiologic and aging research, as studies report associations between childhood circumstances and health in later life. Research on adults has found lower childhood SES to be associated with poor cognitive and physical health, [6,7] and poor psychosocial functioning in mid-life [8]. It has also been found to be associated with myocardial infarction,[9] cardiovascular disease,[10] adult mortality risk, [11-19] cardiovascular mortality, [20,21] and cardiovascular risk factors[22,23] These studies establish a connection between childhood SES and health in middle age and older age.

However, many of these studies have one or more conceptual or methodological limitations. One is that "childhood" is conceptualized as a single, unvarying time frame. Because SES can change over time, it is important to explore experiences during childhood and adolescence. Another limitation is that most of the studies do not include ethnically diverse samples. With the current attention in the United States (US) on health disparities and the ethnically heterogeneous population, there are critical gaps in our understanding of how childhood circumstances are associated with adult health across diverse populations. There is a need to explore these issues in diverse populations to assure relevance and to enable research on the mechanisms by which racial/ethnic disparities in health occur.

In addition, with a few exceptions, many of these studies used single dimension measures of childhood SES such as father's occupation or educational attainment. It is likely that these are the most common measures due to relative ease of recall. Also, most of these studies were conducted in Europe where occupation is traditionally more tied to lineage and social status. Other single dimension measures that have been used include family size, presence of two parents while child is growing up, housing characteristics, work status of the mother, or marital status of the mother at the time of child's birth [24].

Alwin and Wray suggest that research into how social inequalities emerge over a life course is limited by use of the conventional and single dimension measures of SES [25]. They argue that investigating the life course demands attention to family background and other components of social status. Social status is a broader concept than SES, and includes *ascribed *statuses linked to group membership (e.g. born into a family with resources) and *achieved *statuses gained through opportunity (e.g. had access to good schools, got scholarships, and became a doctor) [25]. Conventional SES measures that do not account for this broader range of concepts may not be adequate to address racial/ethnic disparities in health – but to date, there are no instruments that capture this array of potential concepts. For scholars in the nexus of social science and public health, there is a need to agree upon a conceptualization of social status with particular relevance to health outcomes, as well as a comparably standard instrument to measure socioeconomic status in such a way as to take into consideration other types of status [4].

To further research in this area, conceptualizing SES in childhood as multidimensional would allow for disentangling different aspects of childhood socioeconomic status, perhaps drawing from facets typically attributed to the concept of social status. Expanding the content of measures of childhood SES could advance the field of health disparities research by considering the various mechanisms that generate racial/ethnic disparities in health. As briefly reviewed above, SES is associated with a range of health outcomes from health behaviors to chronic and infectious diseases. Childhood SES has also been linked to general health and functioning measures and specific chronic diseases. In order to expand the conceptualization of SES to further investigate the mechanisms, we elected not to target a specific health outcome. The array of health outcomes associated with SES suggests that it may influence multiple pathways.

This article reports on a concept development study conducted to explore childhood experiences relevant to SES among an ethnically diverse sample. The purpose of this study is to advance the conceptualization of childhood social and economic experiences that would facilitate life course studies of the associations between childhood circumstances and older adult health. Such a concept development study is a necessary step toward development of a measurement tool.

## Methods

To construct an expanded framework of childhood social and economic circumstances relevant for ethnically diverse older adults, we used in-depth interview methods. We used qualitative research methods for two reasons. First, because social and economic circumstances are complex and varied, it is important to gather in-depth information from individuals to identify the most relevant experiences. Second, we needed to find out which childhood events people tended to recall.

In creating our interview guide, we aimed to expand the concept of childhood SES to include a variety of childhood social and economic circumstances, in order to include the breadth of experiences that appear to be relevant to the development of social status over the life course. We reviewed literature that documented associations between childhood circumstances and health in adults and older adults and the literature on conceptualizations of SES relevant to health [3-5]. We also drew from sociologists whose work emphasizes that individuals' socializations and lived experiences differ in terms of social class[26,27]. In particular, we drew from an ethnographic study of the socialization of African American and European American children that examined the habits and practices (e.g. discipline, play, extracurricular activities, interaction with teachers and other adults) of families of different social classes [27].

Interview topics included: 1) childhood home and neighborhood environments; 2) economic circumstances [changes over time, if any]; 3) educational experiences [asked separately about middle school/junior high and high school]; 4) recreation and summer experiences; 5) influences of adults; 6) discrimination; 7) leisure activities; and 8) influence and support of adults. These topics expand conventional conceptualizations of SES by including dimensions of social status, especially ascribed status and achieved status[25]. Questions were open-ended in order to allow participants to describe their experiences in their own words. Here are some examples of the questions that we asked: When you were between the ages of 5 and 18, how many places did you live?; [if more than one], of these places is there one that you remember more clearly than the others? Please describe it? Who lived there with you?; When you were in middle school or junior high, what did you do during your summer vacations?; How old were you when you started to work for pay? What sort of work did you do? How did you get the job? What did you do with the money you earned?

### Sampling

We constructed a purposive sample of older adults from diverse racial/ethnic groups (White, African American, Asian American, and Latino). We recruited participants who live in the San Francisco Bay Area through organizational contacts and personal contacts of the authors (IHY, AS, and EPS), and snowball sampling. Three of the authors (IHY, ALS, and EPS) are investigators with the University of California, San Francisco Center on Aging in Diverse Communities (CADC). CADC projects have involved a variety of community organizations that serve older adults (e.g. senior centers, faith-based organizations, community clinics).

Eligibility criteria included: (a) age 55 or older; (b) self-identified as White, African American or Black, Asian American, or Latino/Hispanic; (c) born in the US or emigrated before age 16 and (d) English-speaking. We aimed to interview 16 women and eight men; this ratio was set in order to reflect the higher proportion of women in older age groups. We aimed to split the sample as evenly as possible across the four racial/ethnic groups, that is sample 5 to 7 people from each racial/ethnic group [28].

### Data Collection

One author (IHY) conducted all interviews, which took place in participants' homes or a location of the participant's choosing. The semi-structured interview format allowed us to modify the questions depending on the participants' responses. Because new topics or concepts were brought up in several of the initial interviews, we adjusted the interview guide to include questions about these topics in subsequent interviews. For example, several participants in early interviews described instances of discrimination in housing and education. In subsequent interviews, we asked participants about discrimination if they did not bring the topic up on their own.

All interviews were conducted in English, lasted between 45 and 90 minutes, and were tape recorded and transcribed verbatim. Participants were paid $20 for the interview. Study procedures were approved by the UCSF Institutional Review Board. Transcripts of the interviews were available within two weeks of the interview. While formal coding of the transcripts began after almost all of the interviews were completed, the interviewer knew from review of the transcripts that there was a range of experiences described in the interviews, that in-depth narratives had been provided and that a sufficient level of saturation had been attained, that is no new content out of the range of previously described experiences was emerging from the interviews. This sense of saturation provided the authors with a sense of completion with regard to sample size.

### Analysis

Two of the authors (IHY and TS) analyzed transcripts by systematically coding text, guided by the interview topics, as well as themes that emerged from the data [29]. All codes were assigned to text blocks using QSR NVivo version 2.0. Our process of data reduction, " the process of selecting, focusing, simplifying, abstracting, and transforming the data" [30] was guided by our intent to construct survey questions that reflected the different domains and could be intelligible to future research participants from diverse backgrounds. Organizing our data was guided by our selection of themes on which to base our survey questions.

Analysis took place in several stages. For the first six transcripts, one author (IHY) read the transcript and assigned codes relating to the topics and questions in the interview guide, as well as other codes that emerged from the data. Based on this initial coding, a preliminary organization of the codes was constructed, loosely grouped together into larger categories or thematic domains. In a second stage, two of the authors (IHY and TS) coded the next 10 transcripts, refining the codes and the thematic domains. In this stage, each author coded each transcript independently, and then together went through each transcript to jointly decide on the coding. Differences were discussed and resolved until there was consensus between the two authors. Discussions during the joint coding meetings also identified emergent topics and themes.

During this phase of data analysis, the authors wrote analytic memos to describe the implications and details of these codes and the larger categories that helped organize the codes [29,31,32]. The analytic memos were the investigators' method of recording emerging themes, similarities and differences of participants/experiences, patterns or trends, common phrases or attitudes, and a tool to query the data (i.e. after coding a number of transcripts, we would identify an emerging theme, write a memo about the theme, take the observation to other transcripts to confirm the presence in another person's experience). The remaining nine transcripts were coded by IHY using the jointly developed coding schematic.

## Results

Our final sample was diverse in terms of race/ethnicity and gender, comprising 25 persons ages 55 to 80 (mean age 63.4 years) who grew up in different regions of the US and Puerto Rico (see Table 1). Based on interview data, participants came from a wide range of economic circumstances. Data that guided our understanding of respondents' economic background included reflections on the families' material circumstances, descriptions of the type of work the parents did, and descriptions of their neighborhoods. In terms of their current socioeconomic status (SES), though we did not intentionally target a range of SES, our group of participants reflected a wide range. For example, the participants' educational attainment ranged from not completing high school to graduate or professional school training (e.g. an attorney, a medical doctor). About 25% of the participants graduated from high school or less. Some people lived in subsidized housing or low-income neighborhoods known for violence and drug-related activities, others lived in middle-income areas, and still others lived in affluent neighborhoods.

**Table 1:** Descriptive information of respondents (n = 25)

Demographic characteristics	Overall N (%)
Sex	
Men	8 (35%)
Women	17 (65%)
Race/Ethnicity	
White	7 (27%)
African American	6 (23%)
Latino	7 (27%)
Asian	5 (23%)
Region or state where grew up primarily	
California	8 (35%)
West (Utah, Arizona)	2 (8%)
Midwest (Minnesota, Michigan, Ohio)	3 (12%)
East (New York, Pennsylvania)	5 (19%)
South (Arkansas, Georgia, Tennessee, Texas, Virginia)	6 (24%)
Puerto Rico	1 (3%)

Based on the analytic process described above, we identified eight thematic domains: home and family circumstances, neighborhood, work and money, potential for advancement through schooling, school quality and content, discrimination, influence and support of adults, and leisure activities (See Table 2). These are each described below.

**Table 2:** Domains and subdomains of childhood experiences based on perspectives of older adults from diverse backgrounds

Home and family circumstances
▪ Moving
▪ Major family events: includes divorce or death of a parent
▪ Household composition
▪ Family economic conditions: description of, how they changed over time, housing tenure
▪ Parents' educational attainment
Neighborhood
▪ Type of Neighborhood
▪ Ethnic composition of neighborhood

Work and money
▪ Work experiences: age began to work
▪ Sources of money
▪ Spending habits: how money was spent (e.g. gave to parents, bought candy, bought clothes)

Potential for advancement through schooling
▪ Parents' attitude toward education
▪ Perception of participant of education as a way to get ahead

Schooling quality and content
▪ Quality of middle and high school
▪ Types of courses in high school: "college prep" or vocational classes at high school

Discrimination
Unfair treatment by teachers or other school staff believed by participants to be based on skin color or language spoken (other than English).

Influence and support of adults
Who and how much support or discouragement provided by adults. Examples are parents, grandparents, aunts or uncles, teachers, and school counselors

Leisure activities
▪ After school activities
▪ Summer
▪ recreation

### Home and family circumstances

This domain includes living situation including moving (and reasons for moving), major family events, household composition, family economic conditions, and parents' educational attainment. There was a range of living situations from staying in one house to moving multiple times. Respondents moved for a variety of reasons. In some cases, their family was renting and when they were able to afford to purchase a home, they moved. In other cases, they moved as the family grew and needed more space. These two circumstances occurred commonly with families that had economic means or whose means were improving over time. For example, a 72-year-old white man growing up in New York City described the apartment building where his family lived and explained that the building was owned by his father's employer. His family was able to live there because the company provided this as a benefit. Later they bought their own home, with more bedrooms. In another example, an African American woman was living with her family in Arkansas. Her father learned of good work opportunities in the shipyards in California, so they moved. An example for families with more limited economic means was a case of a family renting but needing to move because the landlord needed the property.

Respondents also described changes in household composition or family relationships. Household composition – and economic circumstances – changed for some respondents with grandparents moving in as they became frailer or children from another household moving to join the respondents' families. One man described his family's situation as abruptly changing from dealing with a "limited amount of food" to a comfortable middle-class lifestyle. He attributed this improvement to a reduction in the amount of financial aid his father had been providing to his father's extended family.

There was...a very limited amount of food and I think it wasn't until we moved to [town], which happened when I was 18, that our total economic circumstances miraculously changed. So I think my father was helping a lot of his family members in [city] and that's why when we moved to [town] I guess he was helping less and then we had a lot more money. (male, age 67, Latino)

Divorce or death of a parent had a dramatic impact on home and family circumstances. Several women described how they "became the housewife" after the loss of their mothers from divorce or death.

*I was age nine when they got divorced*. [Interviewer: How did your family life change after the divorce?] *Uh, it wasn't easy. I became the housewife, I did all the house cleaning, the laundry, the works. (female, age 55, White)*

### Neighborhood

This domain describes how people talked about their neighborhoods, including who lived in the neighborhood, how well they knew their neighbors, and whether they played with neighborhood kids. Commonly, respondents described their neighborhoods as ''working class'' or ''middle class.''

...working class, lower class neighborhood, a lot of farm workers in the area, because it was an agricultural area so there was a lot of farm workers and mostly lower education kind of neighborhood. (male, age 56, Latino)

Respondents also volunteered information about the ethnic composition of the area. In discussing this, many respondents described their experiences of segregation.

The neighborhood consisted mostly of Chinese. There were very few Caucasians, where we lived with Chinese or Japanese. Back then, you know, there was more discrimination. So the Chinese stayed together in one area. (male, age 74, Asian)

### Work and money

This domain includes the types of jobs, if any, respondents had as children and what they did with their earnings. Working during adolescence was very common regardless of race/ethnicity. Several respondents described working as a means to contribute to the family income.

I started working when I was twelve or younger. I had a paper route. As time went by, I worked with my dad during the war. He helped open up the Officers Club at [town] during the war. They were hard pressed for help because everyone was going into the service. I was young but I went to work with him there, tending bar. (laugh)... I was about fourteen, fifteen at that time. But, before that I had other odd jobs. I had a paper route and other odd things around the neighborhood to get money. (male, age 74, Asian)

Some respondents mentioned experiences of working in a family business without pay.

So when we got to [city] we actually had a maintenance business to clean a lot of the buildings in the [city district] and my dad did it, my brother and I we helped out. So I had my whole section of office buildings that I'd clean off the desk, dump the ashtrays,... cleaning the bathrooms, did all that and..., I was ten, nine, ten, something like that. We worked for about three or four hours in the night, in the evening after working hours. (male, age 56, African American)

People gave a variety of responses when asked how they used their spending money, such as purchasing candy, clothes, music, and going to the movies. Some said that they gave all earned money to their parents (including five or ten cents for running an errand for a neighbor).

Allowances were less common for the Latino, Asian, or African American respondents. Rather than an allowance, if family means permitted, these respondents indicated that if they needed something, they would ask for the money. One African American woman, who did not have an allowance, said that each month on pay day, her mother would take her out for a meal and buy her a small gift.

### School quality and content

This domain refers to the quality of schooling and the type of classes that the respondents described taking in high school. Respondents described the quality of their schooling, the availability of special programs for high-achieving students (akin to what are often currently termed "magnet" schools or programs for the "gifted and talented"), types of courses they were taking (e.g., "college prep") – or not taking if they were not available in their school, and additional schooling such as Chinese language school during the afternoons after conventional school. One example of the quality of education comes from a 59-year-old Asian woman who grew up in New York City. She described how her smaller high school could provide more individualized attention to the students.

The focus was so much on academics there. That was everybody's focus. I think in general people got along well, teachers were good, classes were small, and if there were any problems that arose they would have been dealt with very quickly. Because it was a small school, counselors were available to you easier than if it was a large school. (female, age 59, Asian)

In contrast, several participants mentioned poor teaching.

*Of course we didn't realize that what they were teaching us at [school] was just deportment *[sic] *, they were not teaching us academics at all, very little...(female, age 80, Latina)*

Schools also had reputations that could influence one's future opportunities. One participant described how he could choose one of two local high schools to attend – and that one had a reputation for sending more graduates on to college, so he chose that one.

### Potential for advancement through schooling

This domain refers to descriptions of participants' or their families' views about school and their decisions about school options. This domain focuses on the individuals and their perceptions, rather than schools as institutions. School was often described as an important means of upward mobility, a "ticket out" or the way to do better than one's parents. This was particularly salient in families with limited financial resources, who could not afford private schools or college for their children. One woman from such a family recalled her parents' commitment to their children's education as a key to their upward mobility and self-sufficiency as adults.

.... Both of my parents were determined that their kids were going to have better than them. My mother and my father were the first ones, in their respective families, to graduate high school. So, they were determined that I was going to go to college and my sister was going to go to college....They didn't have the money for it and they never had the money for it. So, what they were doing was getting me the best education that I could get in the public schools and encouraging me to be self-sufficient and that kind of stuff. (female, age 61, White)

A Latino man described how his father, the owner of a small moving company, insisted that all his children go to private school in order to provide the best quality of education his parents could afford.

The idea that school could be a means to advance was common across racial/ethnic groups. Participants living in modest or lower income circumstances, however, were more likely to mention schooling in this light than those whose families had more resources.

### Discrimination

This domain refers to people's descriptions of differential treatment based on their race/ethnicity. One African American woman went to a school outside her neighborhood because she was academically qualified to do so. Her neighbors were primarily African American, but the school student body was not. She noticed that students "quietly" avoided her (and other African American students) or were only friendly to her to a point.

Well, it was just a kind of quiet exclusion. It was very clear from the way that we didn't get included in things that we were from out of the area. We were not a part of their neighborhood and so you really just didn't get invited to do certain things. Like, when we were in the actual school building, in the classroom, everyone was friendly enough but sometimes the friendliness didn't go much beyond the hallway or the change in classes. (female, age 56, African American)

Respondents described how teachers' expectations were associated with their ethnicity. It was not unusual for teachers to comment to a non-white person that they did not have a bright future.

My school was predominantly Mexican...the police, the teacher, the principal and counselors and so on, all those were White, many had negative attitudes towards us, towards Mexicans. I mean negative in the sense that [they] believed, sort of [made] an assumption that...none of us should do well in school and probably could not, just [were not] genetically equipped to do well in school. I think on the unconscious and natural order of things, [they assumed] that we should be prepared for the service occupations.(male, age 67, Latino)

Latinos and Asians described language as a source of tension. Some Spanish-speaking parents insisted that their children speak only Spanish at home, in an effort to stay "authentic." In other cases, Spanish-speaking parents insisted their children speak only English at home in order to promote learning of the dominant language and ease their children's entrance into dominant society. School policy sometimes prohibited speaking languages other than English, even in playgroups. One person said he was frequently "caught" and punished for speaking Spanish; he was at a loss as to how to behave because he could not speak English at the time.

### Influence and support of adults

This domain includes descriptions of the role and influence of adults, both family and non-family. Some respondents received support from school counselors or other adults they happened to encounter. Respondents described how adults helped steer them in helpful ways, such as assigning them to college 'prep' classes in high school or telling them about opportunities for college scholarships.

And maybe one counselor in school, well I didn't know him very well and he had a big impact, I think more than he actually realized. Because when it came down to high school and getting into different tracks, like they used to have the academic track and then the non-academic track and then the vocational track... he said, "well what do you want to do?" and I said, "well..." I didn't want to go into the academic track, because I thought it would be too hard and I didn't really like school that much. But he put me in the academic track anyway, even though I had said I didn't want to go in it.... I don't know if that was legal or illegal on his part, but it actually turned out to be really well for me. (male, age 56, Latino)

Respondents also described how relatives (e.g. uncles or aunts) or friends of their parents were influential by providing them access to experiences to which they might otherwise not have been exposed. They mentioned that aunts or uncles took them to foreign films, on camping trips, or on sightseeing trips to other cities.

### Leisure activities

This domain includes organized and unorganized activities respondents did for fun or recreation, including summertime activities. Respondents' descriptions of leisure and recreational activities ranged from unstructured play (e.g. playing in the streets) to organized activities (e.g. summer bible camp).

Dodge ball and jump rope and hop scotch and roller skating [street name] was the main street and then there were two little small feeder streets off of it. So, the kids came from all three blocks. Just a little bit of everything and hide and seek. I mean everybody knew everybody so we were just all over the place. We would have our little dance things in the middle of the street. We'd put the music on and everybody would get in the street and start dancing. (female, age 56, African American)

Well there were no, not too many playgrounds or anything. There was one, there was one little park in the area that we used to go to.... it had a little kiosk in the middle and we would just kind of hang around there and play. And then we'd go to a nursery, there was a huge nursery outside of town, where they had ditches for irrigation and stuff, it was a nursery that sold trees and plants and things like that. So we would just hang out there and pretend that we were in a jungle. (male, 56, Latino)

One woman reflected that the very concept of "leisure" or play marked differences between her and her peers.

*Q: *Did you think that leisure activities or the things that they [people she met in college who were from 'different' backgrounds] did on their own time were different?

*A: Well, that they had leisure. I think that it was something that was a new idea to me. I don't think that growing up we had a good deal of leisure. Of course, we had Saturday or some Sundays after church but otherwise it was chores, school, homework, or whatever. (female, 59, Asian) *[emphasis in original]

There was some variation by gender concerning leisure and recreational activities. Several women mentioned that their parents prohibited them from going far and that they were assigned lots of household chores; this combination kept them close to home. None of the men mentioned these types of limits on their activities. Play activities seemed to cut across racial/ethnic groups, with respondents in all groups mentioning unstructured activities.

We asked about summer break activities and heard a range of responses. Some respondents stayed close to home and played with friends in the neighborhood. Others attended summer camp for part of the summer. Respondents mentioned taking yearly trips to visit relatives in another part of the country. In a few instances, respondents mentioned sightseeing trips.

## Discussion

This exploratory study has identified several domains of childhood social and economic circumstances not often examined in prior research linking childhood socioeconomic conditions to health in later life. Our data extend traditional conceptualization of childhood SES by 1) illustrating the importance of other experiences such as housing circumstances (owning or renting), and 2) highlighting the domains of discrimination, leisure activities, and influence and support of adults. In the case of housing, current conceptualizations of adult SES have indicated that assets and wealth should be taken into consideration beyond income. Similarly for children, their housing circumstances can be included when ascertaining their economic experiences. The domains of discrimination, leisure activities, and influence and support of adults have not consistently been considered when measuring childhood social and economic circumstances. These additional domains expand the dimensions of childhood SES building on Alwin and Wray's identification of social status as informed by both ascribed and achieved status.

Our data illustrate the "opportunity structures" that were available to respondents while they were growing up. Opportunity structures are an array of institutions, networks, and social and administrative systems (e.g. school, work, and housing) that provide resources for economic advancement [33]. For example, in the home and family circumstances domain, some respondents described their families' upward mobility in terms of moving to bigger houses, better neighborhoods. In terms of education and educational opportunity, the type of school programs, the perceived role of school, and discriminatory treatment were emphasized. Influence and support of adults and leisure activities contributed to both economic and educational circumstances and experiences.

Our data also expand some of the traditional concepts of childhood SES such as parental income, occupation, and education. For example, our data show that for children, "education" has at least two dimensions in addition to grade level completed and grades or marks. One dimension refers to the "schooling quality and content" or the perceived quality of the instruction provided by the teachers and the program or curriculum offered by the school. The other dimension refers to how schooling is perceived by the child and his/her family as a means for advancement.

Another example of how these results enrich the conventional domains of childhood SES is the content on money and work. Most of the older adults we interviewed reported work experiences and the experiences varied from running errands for a neighbor for small change to working in a local shop as a sales assistant. Separate from the sort of work their father did, this domain highlights the individual's own work experiences as a child or adolescent.

In addition to adding depth and breadth to the traditional framework, the data also suggested some potentially interesting gender and racial/ethnic differences. Women mentioned having household responsibilities such as housework or care of siblings, whereas men did not mention these activities. Men's recreational activities were likely to have more geographic range than women's. In some cases, women mentioned being specifically prohibited from spending free time away from home. In terms of racial/ethnic differences, by including a diverse racial/ethnic sample, we learned about experiences of unfair treatment in school and work. The first three respondents brought it up as an important part of their childhoods, including one white woman who was assigned to a school in an African American neighborhood; her father was uncomfortable about her going to that school. Almost all of the non-white respondents mentioned some aspect of discrimination or segregation as being important in their childhoods.

The expanded framework can contribute to an expanded multidimensional measure of childhood SES for older adults in the US. Translated into a survey instrument, we can investigate the associations of a range of childhood circumstances with health in middle-aged and older adults. For example, the domains of home and family circumstances, and influence and support of adults, suggest useful areas to explore in further research. Previous research has reported associations between parental loss or divorce, strained relations with parents, and adult health [34-37]. These studies have not investigated if other adults were present and supportive, perhaps compensating for the loss of a parent due to divorce or death.

An important limitation of this method for expanding the dimensions of childhood SES concerns the phenomenon of autobiographical memory (a form of differential recall bias for epidemiologists) and how people's reports of the past may be altered by subsequent experiences [38-40]. For example, a more successful adult may see their schooling as high quality and report a lot of adult support as a child, whereas a less successful adult may recall poor school quality and encounters with people who hindered their progress. In the future it would be ideal to have ways to externally validate what study subjects report.

Another limitation of our study is the small sample size. For an exploratory study, our sample size is sufficient due to the diversity of the sample and the variation of experiences observed. Qualitative researchers point out that sample size goals depend on the type of research. One researcher suggests that for discerning the essence of experiences, about six participants is recommended[28]. Our respondents were recruited intentionally to be balanced across four major racial/ethnic groups, thus there are a disproportionate number of non-Whites compared to the demographics of the older population of the state. (Figures from the Current Population Survey for 2005 indicate that 61% of the over 50 population are White, 19% are Latino, 12% are Asian, and 6% are African American[41]) As in most qualitative research, participant samples are rarely representative of a larger population. Although we have some data that suggests divergent experiences based on race/ethnicity, we are reluctant to draw firm conclusions without larger numbers of respondents.

Related to the small sample size limitation is the possibility of selection bias. Qualitative researchers generally do not discuss selection bias because they do not seek to represent a larger population per se, but rather through in-depth narrative or "thick description" set out to document experiences, processes, and relationships[42]. Evaluation of bias is made by assessing the contents of the data, whether the identified themes are plausible and whether the connections that the investigators make are reflected in the data and not imposed by the investigators. These quality checks occur when the interpretations are presented to other investigators and to other people who could be part of the study group, in this case older adults.

These interview data have identified an expanded set of dimensions to improve measurement of children's SES that may help us understand older adult's current health status. We are developing a survey measure from these key domains in such a way to ascertain a person's material circumstances (e.g. household income), educational opportunity (e.g. school quality), and psychosocial circumstances (e.g. emotional support). In this way, when we investigate the trajectory of exposure for SES over time, we can uncover a more detailed understanding of the influence of SES on health.
